# Differential Transmission of Old and New World Begomoviruses by Middle East-Asia Minor 1 (MEAM1) and Mediterranean (MED) Cryptic Species of *Bemisia tabaci*

**DOI:** 10.3390/v14051104

**Published:** 2022-05-20

**Authors:** Saurabh Gautam, Habibu Mugerwa, James W. Buck, Bhabesh Dutta, Tim Coolong, Scott Adkins, Rajagopalbabu Srinivasan

**Affiliations:** 1 Department of Entomology, University of Georgia, 1109 Experiment Street, Griffin, GA 30223, USA; sg37721@uga.edu (S.G.); habibu.mugerwa@uga.edu (H.M.); 2 Department of Plant Pathology, University of Georgia, 1109 Experiment Street, Griffin, GA 30223, USA; jwbuck@uga.edu; 3 Department of Plant Pathology, University of Georgia, 3250 Rainwater Road, Tifton, GA 31793, USA; bhabesh@uga.edu; 4 Department of Horticulture, University of Georgia, 3250 Rainwater Road, Tifton, GA 31793, USA; tcoolong@uga.edu; 5 United States Department of Agriculture-Agricultural Research Service, U.S. Horticultural Research Laboratory, Fort Pierce, FL 34945, USA; scott.adkins@usda.gov

**Keywords:** cucurbit leaf crumple virus, midgut, primary salivary glands, sida golden mosaic virus, tomato yellow leaf curl virus

## Abstract

Middle East-Asia Minor 1 (MEAM1) and Mediterranean (MED) are two of the most invasive members of the sweetpotato whitefly, *Bemisia tabaci,* cryptic species complexes and are efficient vectors of begomoviruses. *Bemisia tabaci* MEAM1 is the predominant vector of begomoviruses in open-field vegetable crops in the southeastern United States. However, recently *B. tabaci* MED also has been detected in the landscape outside of greenhouses in Florida and Georgia. This study compared the transmission efficiency of one Old-World (OW) and two New-World (NW) begomoviruses prevalent in the southeastern United States, viz., tomato yellow leaf curl virus (TYLCV), cucurbit leaf crumple virus (CuLCrV), and sida golden mosaic virus (SiGMV) between *B. tabaci* MEAM1 and *B. tabaci* MED. *Bemisia tabaci* MEAM1 efficiently transmitted TYLCV, CuLCrV, or SiGMV, whereas *B. tabaci* MED only transmitted TYLCV. Percent acquisition and retention of OW TYLCV following a 72 h acquisition access period was significantly higher for *B. tabaci* MED than *B. tabaci* MEAM1. In contrast, *B. tabaci* MEAM1 acquired and retained significantly more NW bipartite begomoviruses, CuLCrV or SiGMV, than *B. tabaci* MED. Quantitative analysis (qPCR) of virus DNA in whitefly internal tissues revealed reduced accumulation of CuLCrV or SiGMV in *B. tabaci* MED than in *B. tabaci* MEAM1. Fluorescent in situ hybridization (FISH) showed localization of CuLCrV or SiGMV in the midgut of *B. tabaci* MED and *B. tabaci* MEAM1. However, localization of CuLCrV or SiGMV was only observed in the primary salivary glands of *B. tabaci* MEAM1 and not *B. tabaci* MED. TYLCV localization was observed in all internal tissues of *B. tabaci* MEAM1 and *B. tabaci* MED. Overall, results demonstrate that both *B. tabaci* MEAM1 and *B. tabaci* MED are efficient vectors of OW TYLCV. However, for the NW begomoviruses, CuLCrV and SiGMV, *B. tabaci* MEAM1 seems to a better vector.

## 1. Introduction

Inadvertent introduction of a new species into geographical areas can cause large-scale ecological and economic consequences [1,2]. Insects form the biggest part of all non-native invasive animal species on the planet [3]. Introduced insects can interact with and competitively displace closely related indigenous species, and by serving as vectors, they can significantly alter the dynamics of dormant native or introduced pathogens [4,5,6,7,8,9]. In 2004, *Bemisia tabaci* Gennadius Mediterranean (MED, formerly known as Q biotype), was first detected in the United States in Arizona [10]. *Bemisia tabaci* MED is now present in greenhouses in 23 states in the United States [10,11]. For the first time, in 2016, *B. tabaci* MED was detected outside of the greenhouse in Florida, and more recently it was documented in snap bean fields located in proximity to urban landscapes in Georgia [12,13]. Consequently, the probability of *B. tabaci* MED moving into open crop production systems has increased in the southeastern United States. 

*Bemisia tabaci* Middle East-Asia Minor 1 (MEAM1, formerly known as B biotype) and *B. tabaci* MED are members of the cryptic species complex [14,15,16,17,18,19,20]. In 1980, the introduction of the invasive *B. tabaci* MEAM1 in the southern United States resulted in significant changes in whitefly population dynamics. Unprecedented outbreaks of *B. tabaci* MEAM1 were observed in field-grown row and vegetable crops [21,22,23,24,25]. Due to greater polyphagy and stronger ability to adapt to different environmental conditions, *B. tabaci* MEAM1 quickly displaced the native *B. tabaci* A biotype [26,27]. A few years after the introduction of *B. tabaci* MEAM1 into the southern United States, epidemics of previously unreported whitefly-transmitted viruses were documented in Cucurbitaceae and Solanaceae crops [26,28,29]. Similar patterns of whitefly infestation and whitefly-transmitted virus epidemics have been observed in important vegetable crops in China and Brazil upon invasion and establishment of *B. tabaci* MED and *B. tabaci* MEAM1, respectively [30,31,32,33,34,35]. Likewise, detection of *B. tabaci* MED in the southeastern United States in the landscape/crop fields has raised serious concerns about the transmission dynamics involving prevalent and/or new viruses, especially begomoviruses as they account for 90% of the whitefly-transmitted viruses [36]. *Bemisia tabaci* MED has been shown to have a higher propensity to develop resistance to commonly used insecticides in whitefly management programs in vegetable crops [12,37,38]. Under high insecticide pressure, *B. tabaci* MED can outcompete, *B. tabaci* MEAM1 [39]. In China, Japan, and South Korea, *B. tabaci* MED has begun displacing *B. tabaci* MEAM1 as the predominant invasive *B. tabaci* cryptic species [40,41,42,43]. Studies from China have reported that tomato yellow leaf curl virus (TYLCV) is transmitted more efficiently by *B. tabaci* MED than *B. tabaci* MEAM1, and the recent epidemics of TYLCV in China have been associated with outbreaks of *B. tabaci* MED [44,45].

Begomoviruses are transmitted in a persistent circulative manner by whiteflies in the *B. tabaci* cryptic species complex [46,47]. For successful inoculation upon acquisition, begomoviruses must overcome the midgut (MG) and the primary salivary glands (PSG) barriers [48,49]. Capsid protein is the only known virus-encoded protein that is reported to mediate begomovirus interactions within whiteflies [50]. The amino acids between two conserved regions in the capsid protein, viz., GCEGPCKVQS and LYMACTHASN, seem to influence begomovirus movement in *B. tabaci* [51,52,53]. Begomoviruses with high capsid protein sequence similarity may share similar transmission patterns and could be cryptic-species-specific [52]. For instance, viruses with high capsid protein similarities, viz., tobacco curly shoot virus (TbCSV) and the cotton leaf curl Multan virus (CLCuMuV), were more efficiently transmitted by *B. tabaci* Asia II 1 than by *B. tabaci* MEAM1. This was presumably because both viruses were able to efficiently cross the midgut barrier in *B. tabaci* Asia II 1 than in *B. tabaci* MEAM1 [52]. Other than the virus capsid protein, many vector factors might also be involved in begomovirus interactions within whiteflies [53,54,55]. However, those remain poorly understood. Depending on the interactions between virus capsid protein, endosymbionts and/or vector factors, different *B. tabaci* cryptic species have been documented to transmit viruses with varying efficiencies. For instance, inefficient transmission of TYLCV by *B. tabaci* MED in comparison with *B. tabaci* MEAM1 is reported to be dependent on the presence of an endosymbiotic bacterium (*Hamiltonella*) in *B. tabaci* MEAM1 and its absence in *B. tabaci* MED populations [56]. 

Tomato (*S. lycopersicum*), squash (*Cucurbita pepo* L.), and common (snap) bean (*Phaseolus vulgaris* L.) are important summer crops in the southeastern United States. Tomato-infecting tomato yellow leaf curl virus (TYLCV), squash- and snap-bean-infecting cucurbit leaf crumple virus (CuLCrV), and snap-bean-infecting sida golden mosaic virus (SiGMV) are important whitefly-transmitted begomoviruses in Georgia and Florida. TYLCV is a monopartite Old-World (OW) begomovirus that was introduced into the southern United States in the 1990s [57]. CuLCrV and SiGMV are bipartite New-World (NW) begomoviruses that are endemic to the Americas [58,59]. Currently, under field conditions, TYLCV, CuLCrV, and SiGMV are transmitted by *B. tabaci* MEAM1 [13]. To assess the possible impact of *B. tabaci* MED on virus epidemics, the current study compared the transmission of TYLCV, CuLCrV, or SiGMV by *B. tabaci* MED and *B. tabaci* MEAM1. This study also assessed the acquisition and retention (for 28 days) of TYLCV, CuLCrV, or SiGMV by *B. tabaci* MED and *B. tabaci* MEAM1. Furthermore, this study conducted quantitative (qPCR) and qualitative (fluorescence in situ hybridization, FISH) investigations to quantitate and localize TYLCV, CuLCrV, or SiGMV within *B. tabaci* MED and *B. tabaci* MEAM1 internal tissues. Lastly, based on the earlier studies that reported begomovirus transmission using *B. tabaci* MED, a capsid protein alignment was made and examined with the goal of identifying differences in the capsid proteins of *B. tabaci* MED transmissible and non-transmissible begomovirus species.

## 2. Materials and Methods

### 2.1. Plants, Virus Inoculum Sources, and B. tabaci

Tomato (*S. lycopersicum* L. cv. Florida 47, Harris Moran Seeds Company, Davis, CA, USA), yellow summer squash (*C. pepo* L. cv Gold Star F1 hybrid, Syngenta^®^, Willimington, DE, USA), and prickly sida (*Sida spinosa* L., from field-collected plants) seeds were planted in 1 L plastic pots using Grower Mix (Asb Greenworld, West Point, VA, USA). Plants were maintained in a greenhouse (25–30 °C, 14 h L:10 h D) in insect-proof cages (Megaview Science Co., Taichung, Taiwan) with 5 plants per cage. Plants were fertilized every alternate week with slow-release granular fertilizer, Osmocote (Scotts Miracle-Gro products, Inc., Marysville, OH, USA) as per label recommendations. The TYLCV isolate was collected from a commercial tomato farm located in Montezuma (Macon County, GA, USA) in 2009 and maintained since on TYLCV-susceptible tomato variety Florida 47 through repeated inoculations of three- to four-week-old tomato plants using viruliferous *B. tabaci* MEAM1. The CuLCrV isolate used in this study was collected from infected yellow summer squash plants from Tifton, GA, in 2016 and maintained since on susceptible yellow summer squash plants through repeated inoculations of two- to three-week-old squash plants using viruliferous *B. tabaci* MEAM1. The SiGMV isolate was collected from infected prickly sida plants growing amidst the natural vegetation at the UGA Horticulture Hill Farm in Tifton (Tift County, GA, USA) in 2018 and since maintained on prickly sida plants through repeated inoculations of four- to six-week-old prickly sida seedlings using viruliferous *B. tabaci* MEAM1 [59].

*Bemisia tabaci* MEAM1 (Genbank accession number: MN970031) used in the present study was collected in 2009 from a cotton farm in Tifton Georgia and since reared on cotton plants in whitefly-proof cages in the greenhouse at above-stated conditions. *Bemisia tabaci* MED (GenBank accession number: MZ469725) was collected from poinsettia plants from a nursery located in North Georgia, USA by Dr. Ronald Oetting, UGA. Since 2017, a *B. tabaci* MED colony has been maintained on cotton plants in whitefly-proof cages in the greenhouse at the above-stated conditions. Every alternate month, five to ten individuals from each colony were tested for the purity of the colonies by partially sequencing the *mtCOI* gene using universal COI primers C1- J-2195 and L2-N-3014 [60,61].

### 2.2. Bemisia tabaci Fitness on Tomato, Squash, and Prickly Sida Plants

To assess host suitability, life-history parameters (fecundity, egg–adult survival, developmental time) of *B. tabaci* MEAM1 and *B. tabaci* MED on three plant species (tomato, squash, and prickly sida) were measured. Three two-true leaf stage plants were placed in insect-proof cages in the greenhouse and maintained at the above-stated conditions. Using a clip cage (36.5 mm × 25.4 mm × 9.5 mm), two pairs of recently emerged (up to 48 h old) *B. tabaci* MEAM1 or *B. tabaci* MED adults were attached to the abaxial side of a leaf on each plant and provided with a 48 h oviposition access period (OAP). Post OAP, whitefly adults were removed, and total number of eggs laid were recorded under the dissecting microscope at 10× magnification (MEIJI TECHNO, Santa Clara, CA, USA). Using a 0.1 mm diameter micro pin mounted on a chopstick, the number of eggs per cage were adjusted to five. Cages were monitored daily at ~10 AM until adult emergence. Egg to adult survival and developmental time from egg to adult of *B. tabaci* were recorded. The experiment was conducted three times (*n* = 45 for each whitefly and plant combination). To assess fecundity, a pair of *B. tabaci* MEAM1 or *B. tabaci* MED whitefly adults (up to 24 h old) that developed on tomato, squash, and prickly sida plants were clip caged to two-to-three true leaf stage tomato, squash, and prickly sida plants. Whitefly adults were transferred to new clip cages every fifth day for the next 15 days. Egg-bearing leaves were excised and numbers of eggs were enumerated under a dissecting microscope (10×). Each treatment had 10 replications (10 clip cages), and the experiment was conducted three times (*n* = 30 for each treatment).

### 2.3. Virus Detection 

TYLCV infection status of tomato plants and viruliferous whiteflies was determined through endpoint PCR performed using the primers and conditions described earlier by Legarrea et al. (2015) [62]. Briefly, 5 μL GoTaq Green Mastermix buffer (2×) (Promega, Madison, WI, USA) was combined with primers C2-1159 and C2-1853 (final concentration 0.5 μM) (Table 1), 20 ng DNA, and nuclease-free distilled water for a final volume of 10 μL. PCR was performed using a T-100 thermocycler (Bio-Rad, Hercules, CA, USA). An initial denaturation step (2 min at 95 °C) was followed by 40 cycles of 95 °C for 1 min, 58 °C for 1 min, and 72 °C for 1 min, and a final extension of 72 °C for 10 min.

CuLCrV or SiGMV infection status in infected plants and viruliferous whiteflies was determined through endpoint PCR using the primers and conditions described earlier by Gautam et al. (2020) and Gautam (2019), respectively [59,63]. Briefly, for CuLCrV, 5 μL GoTaq Green Mastermix buffer (2×) was combined with primers 3FAC3 and 5RAC1 (final concentration 0.5 μM) (Table 1), 20 ng DNA, and nuclease-free distilled water for a final volume of 10 μL. PCR was performed using a T-100 thermocycler. An initial denaturation step (2 min at 95 °C) was followed by 40 cycles of 95 °C for 1 min, 54 °C for 1 min, 72 °C for 1 min, and a final extension of 72 °C for 10 min. The presence of SiGMV was tested using primers SiGMVF and SiGMVR (final concentration 0.5 μM) (Table 1). The primers were combined with 20 ng DNA and nuclease-free distilled water for a final volume of 10 μL. PCR was performed using a T-100 thermocycler. An initial denaturation step (2 min at 95°C) was followed by 40 cycles of 95 °C for 1 min, 58 °C for 1 min, and 72 °C for 1 min, and a final extension of 72 °C for 10 min. PCR products were run on 1% agarose gel containing GelRed at 0.5 μg/mL (Biotium, Fremont, CA, USA) and observed under UV light. 

### 2.4. Virus Quantitation

For virus quantitation in samples of infected plants or viruliferous whiteflies, extracted total genomic DNA (plants 10 ng or whiteflies 20 ng) was subjected to qPCR. TYLCV accumulation in samples was determined using conditions described earlier by Legarrea et al. (2015) [62]. Briefly, primers TYLC-C2-For and TYLC-C2-Rev (final concentration 0.25 μM) were combined with 6.25 μL GoTaq qPCR Master Mix (2×) (Promega, Madison, WI, USA), DNA (plants 10 ng or whiteflies 20 ng), and nuclease-free water for a final volume of 12.5 μL. Quantitative PCR was performed in Mastercycler ep realplex^4^ (Eppendorf, Hauppauge, NY, USA) with the following conditions: an initial denaturation step (2 min at 95 °C) followed by 40 cycles of denaturation (15 s at 95 °C) and a combined step of annealing and extension at 65 °C for 20 s. Finally, melting curve analysis was conducted to evaluate the specificity of the fluorescence signal. Absolute virus copy numbers were estimated using the procedure described by Legarrea et al. (2015) [62].

CuLCrV or SiGMV accumulation in infected plants or viruliferous whiteflies was determined using the conditions described earlier by Gautam et al. (2020) and Gautam (2019), respectively [59,63]. Primers CuLCrV-QF and CuLCrV-QR were used for CuLCrV quantitation, and SiGMV-QF and SiGMV-QR were used for SiGMV quantitation (Table 1). Primers, DNA, master mix, and water were used in the same proportions as described above for TYLCV. For CuLCrV, cycling parameters were as follows: 95 °C for 2 min followed by 40 cycles of 95 °C for 1 min, 63 °C for 15 s, and 72 °C for 20 s. Cycling parameters for SiGMV included an initial denaturation step (2 min at 95 °C) followed by 40 cycles of 95 °C for 15 s, 63 °C for 10 s, and 72 °C for 20 s. Melting curve analysis was performed to evaluate the specificity of the fluorescence signal, and absolute virus copy numbers were estimated using the procedure described earlier by Legarrea et al. (2015) [62].

### 2.5. TYLCV, CuLCrV, or SiGMV Transmission by B. tabaci MEAM1 and B. tabaci MED

Virus inoculum sources were maintained in susceptible plants through viruliferous *B. tabaci* MEAM1-mediated transmission. Therefore, to avoid contamination of *B. tabaci* MEAM1 in *B. tabaci* MED transmission assays, insect-free virus inoculum sources were generated. To generate insect-free TYLCV-infected tomato, CuLCrV-infected squash, or SiGMV-infected prickly sida plants, viruliferous *B. tabaci* MEAM1 were obtained by providing whiteflies a 72 h acquisition access period (AAP) on infected plants (TYLCV-infected tomato, CuLCrV-infected squash, or SiGMV-infected prickly sida). Using clip cages, viruliferous *B. tabaci* MEAM1 (~100/plant) was attached to the first true leaves of a non-infected two-true-leaf-stage plants (tomato, squash, or prickly sida). Individual plants with clip cages were placed in insect-proof cages under the conditions described above. One week after removing *B. tabaci* from inoculated plants, all leaves from the inoculated plants were excised, and plants without leaves were transferred to new insect-free cages. Three weeks post leaf excision, infection in the emerging symptomatic foliage was confirmed through endpoint PCR (described above). To further ensure that no whiteflies were present on the plants, the foliage of insect-free virus-infected plants was carefully examined for whitefly adults, nymphs and/or eggs using a hand lens under a fluorescent white light lamp. 

Viruliferous whiteflies used for transmission studies were obtained by releasing 1500 *B. tabaci* MEAM1 or *B. tabaci* MED for 72 h (AAP) on the insect-free infected plants (TYLCV-infected tomato, CuLCrV-infected squash, or SiGMV-infected prickly sida). Following the AAP, 20 whiteflies were randomly collected, and total genomic DNA from individual whiteflies was extracted in 50 μL InstaGene Matrix containing six percent Chelex resin (Bio-Rad, Hercules, CA). Extracted total genomic DNA (20 ng) was subjected to endpoint PCR using the primers and conditions described above.

Batches (10 × 3) of viruliferous *B. tabaci* MEAM1 or *B. tabaci* MED for which at least 60% of individuals tested positive via PCR analysis were used for transmission studies (*B. tabaci* MEAM1 (TYLCV: 90–100%; CuLCrV: 70–90%; SiGMV: 80–90%); *B. tabaci* MED (TYLCV: 95–100%; CuLCrV: 60–80%; SiGMV: 65–85%)). Using a clip cage, viruliferous whiteflies (100 adults/plant) were attached to the first true leaf of a two-leaf stage non-infected plant (tomato, squash, or prickly sida plants). Plants were then held in insect-proof cages in the greenhouse under conditions described above. After a 5-day inoculation access period (IAP), plants were treated with the insecticide, Admire pro^®^ (imidacloprid, Bayer CropScience, Research Triangle Park RTP, NC) as per label recommendations via soil drenching. Four weeks later, the topmost leaf sample (100 mg) was excised and surface-sterilized using a six-step surface sterilization protocol [64,65]. Using a squeeze bottle, collected leaf samples were washed in sterile distilled water, followed by 30 s rinse in 1% bleach, followed by a 30 s wash in 70% ethanol, and three more rinses with sterile distilled water. Water from the last rinsate was collected in a sterile 1.5 mL centrifuge tube. The surface-sterilized leaf tissues were used for DNA extraction. Total genomic DNA was extracted with the GeneJET Plant Genomic Purification Kit (ThermoFisher Scientific, Waltham, MA, USA). Virus infection status in plants was determined through end-point PCR using conditions and programs described above, and the virus was quantitated in infected plant samples using the qPCR protocols described above. Each treatment had 10 replications and the experiment was conducted three times (*n* = 30). Surface sterilization and PCR analysis of water from the last rinsate eliminated potential false positives that may have arisen due to residual virus and/or viral DNA-laden honeydew secreted by viruliferous whiteflies on the samples. Prior to the transmission studies, the efficiency of surface sterilization in removing the residual honeydew sticking on the samples was confirmed with TYLCV-infected tomato plants and *B.tabaci* MEAM1 following TYLCV acquisition (Supplementary Material, Table S1).

### 2.6. Retention of TYLCV, CuLCrV, or SiGMV by B. tabaci MEAM1 and B. tabaci MED

*Bemisia tabaci* MEAM1 or *B. tabaci* MED adults were allowed to feed on insect-free non-infected or virus-infected plants for 72 h. Whiteflies were then transferred to four-week-old cotton plants. Cotton is a non-host for all three viruses (Supplementary Material Table S2). Twenty whitefly adults were randomly collected from the cotton plants at five separate time intervals (72 h, 7 d, 14 d, 21 d, and 28 d). After surface sterilization, total genomic DNA from individual whiteflies was extracted using the protocol described above. TYLCV, CuLCrV, or SiGMV presence and accumulation at each interval were estimated using endpoint PCR and qPCR, respectively. Each treatment had 20 whiteflies. The experiment was conducted three times (*n* = 60 for each treatment). 

### 2.7. TYLCV, CuLCrV, or SiGMV Localization and Accumulation in B. tabaci MEAM1 and B. tabaci MED Tissues

*Bemisia tabaci* MEAM1 and *B. tabaci* MED (up to 48 h old), were transferred onto insect-free infected or non-infected plants (TYLCV-infected or non-infected tomato, CuLCrV-infected or non-infected squash, or SiGMV-infected or non-infected prickly sida). Whiteflies were allowed to feed on infected or non-infected plants for 72 h, after which whiteflies were transferred to four-week-old cotton plants. After 72 h, twenty individual whiteflies from cotton were tested for the presence of the virus. Feeding on virus non-host cotton ensured removal of free-floating virus particles from whiteflies midgut lumen. Using an aspirator, viruliferous or non-viruliferous whiteflies were collected in 10 mL plastic vials and chilled on ice. Individual adult whiteflies were collected with the help of a fine paint brush, then using fine-tip forceps (Dumont Tweezer, Style 5, Electron Microscopy Science, Hatfield, PA, USA) and a 0.1 mm diameter micro pin (Roboz Surgical Instrument, Gaithersburg, MD, USA) mounted on a chopstick, the midgut (MG) and primary salivary glands (PSG) were dissected on a glass slide with a single concave depression cavity in saline buffer (200 μL). The fluorescence in situ hybridization (FISH) procedure followed the protocols described by Kliot et al. 2014 [66]. Briefly, dissected organs were fixed using Carnoy’s fixative (VWR, Monroeville, PA, USA). Following fixation, organs were washed with hybridization buffer (20 mM Tris-HCl pH 8.0, 0.9 M NaCl, 0.01% (*w*/*v*) sodium dodecyl sulfate, and 30% (*v*/*v*) formamide). After washing, specimens were hybridized overnight at 4°C in 500 μL hybridization buffer supplemented with 10 pmol of Cy3-labeled fluorescent oligonucleotide probes. After 12 h, organs were transferred using a custom-made eyelash brush into 30 μL of hybridization buffer containing DAPI (0.1 mg/mL in 1 × PBS, Gene TEX, CA) on a new glass slide. After 5 min, organs were gently covered with a glass cover slip, sealed with Permount (Fisher Scientific, Waltham, MA, USA), and observed under an Olympus BX 60 fluorescence microscope (Olympus Corporation of the Americas, Center Valley, PA). TYLCV was localized using the TYLCV-probe [Cy3 5′-GGAACATCAGGGCTTCGATA-3] reported earlier by Kliot et al. (2014) [66]. The DNA probes, namely CuLCrV-Probe: [Cy3 5′-GCCGAAGCGCGATGCCCCAT-3′] and SiGMV-Probe: [Cy3 5′-CAAGGCCTCTGAATGGGTAA-3′] specific to the DNA-A of CuLCrV and SiGMV were designed using primer3 software [67]. 

For virus quantitation, MG, hemolymph, and PSG from 25 whiteflies were obtained for each treatment. MG and PSG were dissected using the procedure described above, and hemolymph was collected using the procedure described earlier by He et al. [68]. After dissection, MG, hemolymph, and PSG collected from five whiteflies (belonging to same treatment) were pooled and treated as one experimental replicate. A total of five experimental replicates were generated per treatment. Total DNA from samples was extracted using the procedure described above for individual whiteflies. DNA was subjected to qPCR analysis using the procedure described above. Each treatment had five replications. The experiment was conducted two times (*n* = 10).

### 2.8. Comparison of Capsid Protein Sequences of B. tabaci MED Transmissible and Non-Transmissible Begomoviruses

A total of 17 begomoviruses for which *B. tabaci* MED-mediated transmission studies have been conducted prior to the current study were identified (Table 2). Capsid protein sequences of the 17 viruses were aligned with the capsid protein sequences of TYLCV, CuLCrV, and SiGMV isolates used in the current study. The sequences were aligned in Geneious Prime, and aligned sequences were visualized in the Espript 3.0 [69,70].

### 2.9. Statistical Analyses

Data analyses were performed in R version 3.4.2 [82]. Data for egg–adult survival (dead vs. survived) were analyzed using a generalized mixed effect model in the Lme4 package using *glmer* function and setting the family argument as binomial [83]. Within-model treatments were considered as the fixed effect and replications as the random effect. ANOVA was run using the function *Anova* in the package car [84]. Post hoc testing was performed using *glht* function with Tukey contrast in the multcomp package [85]. The median developmental time from egg to adult was analyzed by a non-parametric Wilcoxon rank-sum test (Mann–Whitney U test). Data on oviposition were analyzed using the linear mixed effect model (*lmer* function) in the Lme4 package with treatments as the fixed effect and replications as the random effect [83]. ANOVA was run on the model using the function *anova* in the lmerTest package and post hoc testing was performed using the multcomp package [85,86]. Percent infection in plants (infected vs. non-infected) was evaluated using a generalized mixed-effect model in the lme4 package using the procedure described above [83]. One-way ANOVA and a repeated measures ANOVA were used to compare percent infection and virus retention in *B. tabaci* MEAM1 and *B. tabaci* MED at different time intervals. Percent infection and virus accumulation were analyzed using a generalized mixed-effect model and a linear-mixed effect model, respectively. Virus accumulation in whitefly adults and tissues was analyzed using a linear mixed-effect model. Statistical differences were considered significant at *p* < 0.05.

## 3. Results

### 3.1. Bemisia tabaci Performance on Tomato, Squash, or Prickly Sida Plants

Life history parameters between *B. tabaci* MEAM1 and *B. tabaci* MED developing on each host plant did not vary significantly (Table 3). Egg to adult survival, developmental time, and fecundity on tomato, squash, or prickly sida plants were not significantly different between *B. tabaci* MEAM1 and *B. tabaci* MED (Table 3). Furthermore, no significant differences were observed in the fecundity of *B. tabaci* MEAM1 and *B. tabaci* MED on tomato, squash, or prickly sida plants (Table 3). 

### 3.2. TYLCV, CuLCrV, or SiGMV Transmission and Quantitation

**Figure 1:**
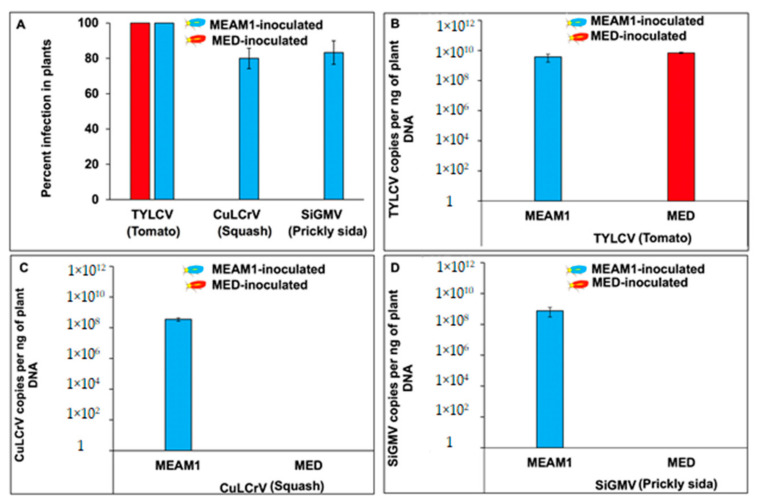
Percent virus infection and virus accumulation in plants inoculated with viruliferous *B. tabaci* MEAM1 and *B. tabaci* MED. (**A**), Bars with standard errors indicate percent TYLCV, CuLCrV, or SiGMV infection in tomato, squash, or prickly sida plants, respectively, inoculated by viruliferous MEAM1 or MED whiteflies. Viruliferous MEAM1 or MED whiteflies were obtained after 72 h AAP on TYLCV-infected tomato, CuLCrV-infected squash, or SiGMV-infected prickly sida plants. Viruliferous *B. tabaci* MEAM1 and *B. tabaci* MED adults were provided with a five-day inoculation access period (IAP) on non-infected two-true-leaf-stage tomato, squash, or prickly sida plants. Four weeks post-inoculation, infection status was confirmed via endpoint PCR. (**B**), Bars with standard errors represent average number of TYLCV copies per ng DNA in tomato plants. (**C**), Bars with standard errors represent average number of CuLCrV copies per ng DNA in squash plants. (**D**), Bars with standard errors represent average number of SiGMV copies per ng DNA in prickly sida plants. Y-axis on panels (**B**–**D**) is represented in logarithmic scale. Significant differences between means were separated with Tukey’s HSD posthoc test at α = 0.05.

Percent transmission of TYLCV did not differ between MEAM1 and MED whiteflies (*χ*^2^_1,58_ = 0; *p* = 1). Furthermore, TYLCV accumulation did not differ between tomato plants inoculated by viruliferous *B. tabaci* MEAM1 or *B. tabaci* MED (*F*_1,28_ = 2.15; *p* = 0.75) (Figure 1A,B). However, CuLCrV transmission percentages by MEAM1 and MED whiteflies differed significantly (*χ*^2^_1,47_ = 24.04; *p* < 0.001). More than 80% of CuLCrV infection was observed in squash plants when inoculated with viruliferous *B. tabaci* MEAM1. In contrast, none of the squash plants inoculated with viruliferous *B. tabaci* MED were infected with CuLCrV (Figure 1A,C). Similarly, SiGMV percentage transmission differed significantly between *B. tabaci* MEAM1 and *B. tabaci* MED (*χ*^2^_1,56_ = 29.14; *p* < 0.001). More than 80% of SiGMV infection was observed in the prickly sida plants when inoculated with viruliferous *B. tabaci* MEAM1. In contrast, none of the prickly sida plants inoculated with viruliferous *B. tabaci* MED were infected with SiGMV (Figure 1A,D). 

### 3.3. Retention of TYLCV, CuLCrV, or SiGMV by B. tabaci MEAM1 and B. tabaci MED

**Figure 2:**
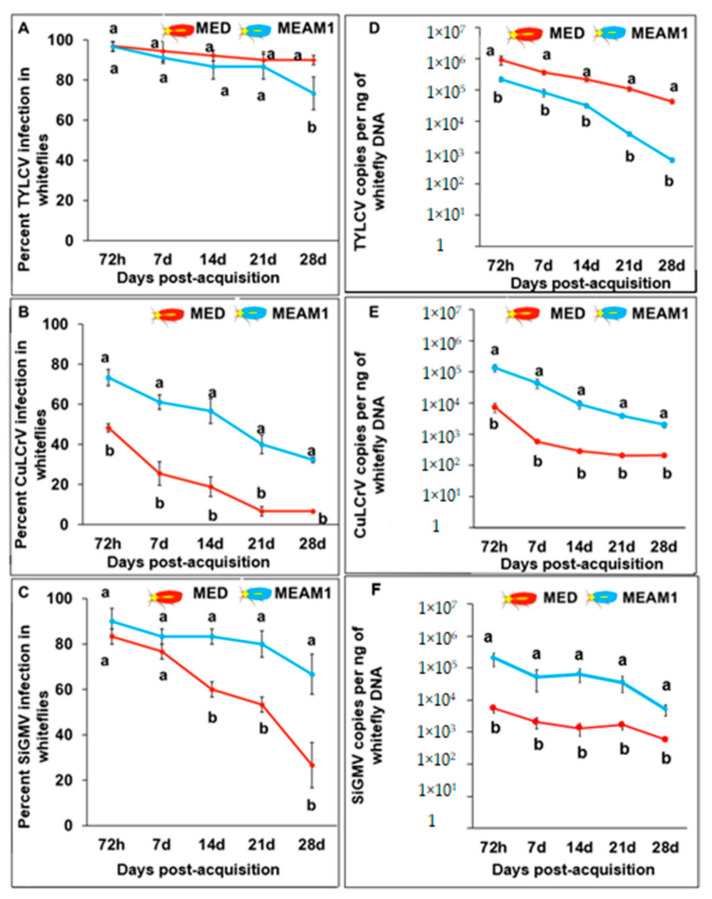
Retention of TYLCV, CuLCrV, or SiGMV by *B. tabaci* MEAM1 and *B. tabaci* MED. Values are means ± SE. Means with different letters are significantly different as separated with Tukey’s HSD post hoc test at α = 0.05. Percent infection in *B. tabaci* adults (MEAM1 and MED) collected from virus non-host cotton plants at five time intervals (72 h, 7 d, 14 d, 21 d, and 28 d) after a 72 h AAP on (**A**), TYLCV-infected tomato plants, (**B**), CuLCrV-infected squash plants, or (**C**), SiGMV-infected prickly sida plants. Virus accumulation in *B. tabaci* adults (MEAM1 and MED) collected from cotton plants at five-time intervals (72 h, 7 d, 14 d, 21 d, and 28 d) after a 72 h AAP on (**D**), TYLCV-infected tomato plants, (**E**), CuLCrV-infected squash plants, or (**F**), SiGMV-infected prickly sida plants. Y-axis is shown in a logarithmic scale in panels (**D**–**F**).

When viruliferous whitefly adults were transferred to virus non-host cotton, the percentages of viruliferous (TYLCV, CuLCrV, or SiGMV) whiteflies decreased over time as indicated by endpoint PCR (Figure 2A–C). At the end of the fourth week, approximately 73% of *B. tabaci* MEAM1 tested positive for TYLCV, 33% *B. tabaci* MEAM1 tested positive for CuLCrV, and 66% *B. tabaci* MEAM1 tested positive for SiGMV (Figure 2A–C). However, the percentages of viruliferous *B. tabaci* MED over five time intervals was virus-dependent. TYLCV incidence in *B. tabaci* MED did not decline gradually with time; at the end of the fourth week, about 90% of *B. tabaci* MED tested positive for TYLCV (Figure 2A). The percentage of *B. tabaci* MED that tested positive for CuLCrV dropped to 7% by the end of the fourth week (Figure 2B). Similarly, the percentage of *B. tabaci* MED that tested positive for SiGMV dropped to 27% by the end of the fourth week (Figure 2C). 

When viruliferous whiteflies were transferred to virus non-host cotton, data from qPCR revealed that the amounts of TYLCV in *B. tabaci* MEAM1 and *B. tabaci* MED that had fed on TYLCV-infected tomato decreased gradually with time (Figure 2D). The amounts of CuLCrV or SiGMV also declined gradually in *B. tabaci* MEAM1. However, in *B. tabaci* MED, there was a sharp decline in the amount of CuLCrV or SiGMV after 72 h. *Bemisia tabaci* MED retained more TYLCV than *B. tabaci* MEAM1 at every time interval (*F*_9,528_ = 8.69; *p* < 0.001) (Figure 2D). However, *B. tabaci* MEAM1 retained significantly more CuLCrV (*F*_9,228_ = 10.58; *p* < 0.001) (Figure 2E) and SiGMV (*F*_9,398_ = 20.08; *p* < 0.001) than *B. tabaci* MED at every time interval (Figure 2F).

### 3.4. TYLCV, CuLCrV, or SiGMV Localization and Accumulation in B. tabaci MEAM1 and B. tabaci MED Tissues

**Figure 3:**
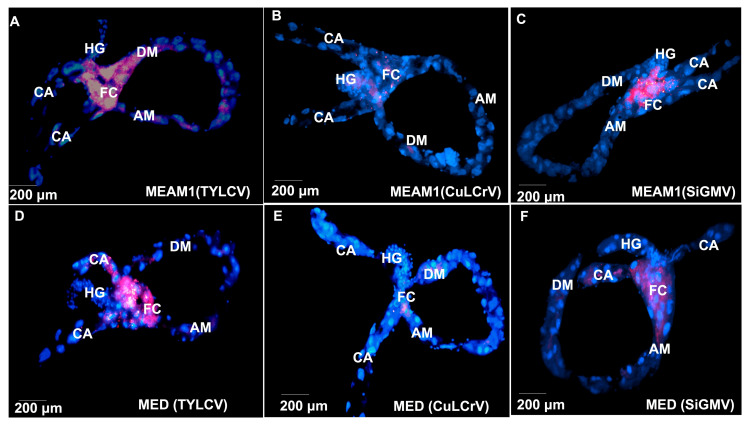
TYLCV, CuLCrV, or SiGMV localization in the midgut dissected from *B. tabaci* adults (MEAM1 or MED) that fed on TYLCV-infected tomato, CuLCrV-infected squash, or SiGMV-infected prickly sida. FISH signals from Cy3-labeled virus-specific probe (red) and DAPI stained nuclei (blue) as seen from Olympus BX 60 fluorescence microscope at 200× magnification using Cy3 and DAPI filter cubes. FC: filter chamber; DM: descending midgut; AM: ascending midgut; CA: caeca; and HG: hindgut. Midgut of *B. tabaci* MEAM1 following (**A**), TYLCV acquisition, (**B**), CuLCrV acquisition and (**C**), SiGMV acquisition. Midgut of *B. tabaci* MED following (**D**), TYLCV acquisition, (**E**), CuLCrV acquisition, and (**F**), SiGMV acquisition.

**Figure 4:**
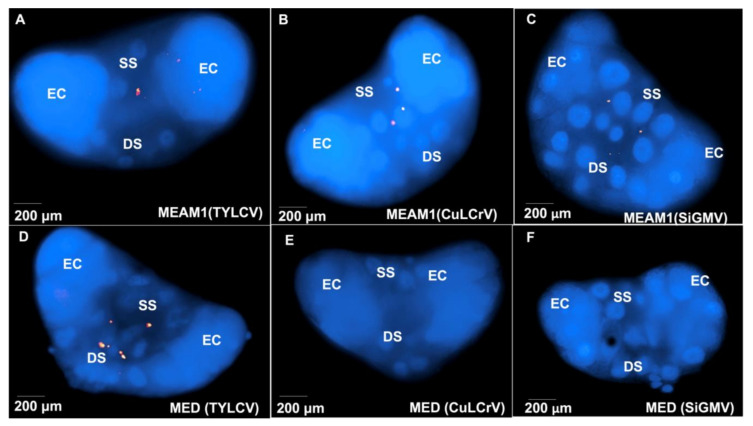
TYLCV, CuLCrV, or SiGMV localization in the primary salivary glands (PSG) dissected from *B. tabaci* adults (MEAM1 and MED) that fed on TYLCV-infected tomato, CuLCrV-infected squash, or SiGMV-infected prickly sida. FISH signals from Cy3-labeled virus-specific probe (red) and DAPI stained nuclei (blue) as seen from Olympus BX 60 fluorescence microscope at 400× magnification using Cy3 and DAPI specific filter cubes. DS—ductal section of the central region; EC—end cap; SS—secretory section of the central region. PSG of *B. tabaci* MEAM1 following (**A**), TYLCV, (**B**), CuLCrV, or (**C**), SiGMV acquisition. PSG of *B. tabaci* MED following (**D**), TYLCV, (**E**), CuLCrV, or (**F**), SiGMV acquisition.

Probes for TYLCV, CuLCrV, or SiGMV hybridized in viruliferous whitefly tissues. FISH revealed that TYLCV, CuLCrV, or SiGMV in *B. tabaci* MEAM1 and *B. tabaci* MED localized in the midgut (Figure 3). Most hybridization was localized within the filter chamber (Figure 3). There was some localization in the descending and ascending midgut as well. FISH signals for TYLCV were detected in *B. tabaci* MEAM1 and *B. tabaci* MED PSG (Figure 4A,D). However, CuLCrV or SiGMV localization was observed in the PSG of *B. tabaci* MEAM1 alone and not in *B. tabaci* MED (Figure 4B,C,E,F). In *B. tabaci* MEAM1, TYLCV, CuLCrV, or SiGMV were primarily localized in the secretory section of the central region of PSG. No virus localization was detected in *B. tabaci* MEAM1 or *B. tabaci* MED tissues feeding on non-infected tomato, squash, or prickly sida plants. 

**Figure 5:**
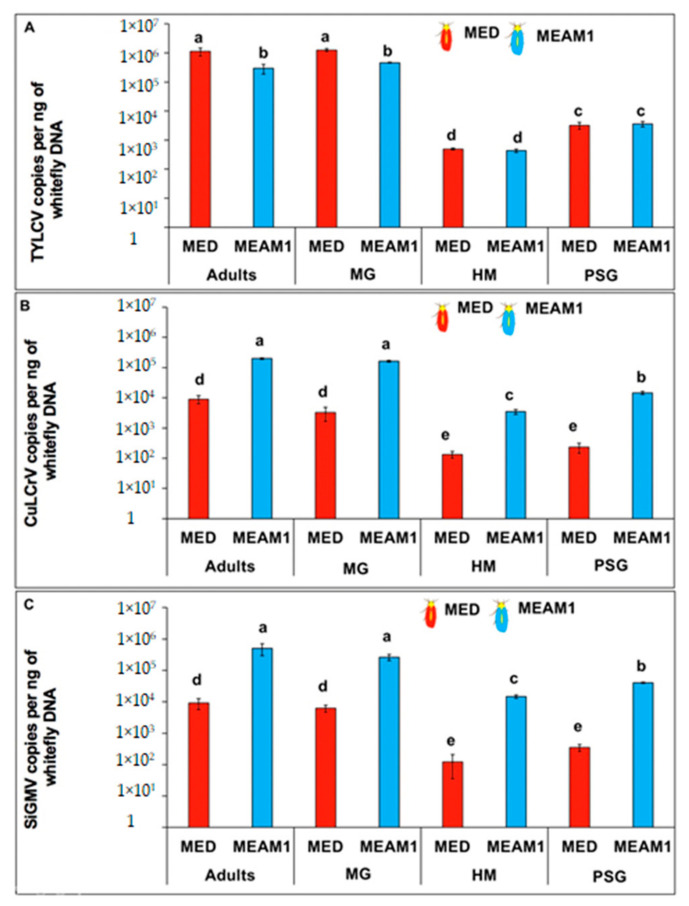
Virus accumulation in *B. tabaci* adults and tissues. Bars with standard errors represent mean virus copy numbers per ng of total DNA within whole adults, midguts (MG), hemolymph (HM), and primary salivary glands (PSG) of *B. tabaci* MEAM1 and *B. tabaci* MED adults following a 72 h AAP on (**A**) TYLCV-infected tomato plants, (**B**) CuLCrV-infected squash plants, or (**C**) SiGMV-infected prickly sida plants after a 72 h gut clearing. Y-axis is represented in a logarithmic scale and significant differences between means were analyzed using Tukey’s test at α = 0.05.

TYLCV accumulation in *B. tabaci* MED and *B. tabaci* MEAM1 adults and tissues differed significantly (*F*_5,54_ = 19.65; *p* < 0.001) (Figure 5A). More TYLCV accumulated in *B tabaci* MED adults and in their MG in comparison with *B. tabaci* MEAM1 adults and in their MG. However, TYLCV accumulation did not differ between hemolymph and PSG of *B. tabaci* MEAM1 and *B. tabaci* MED. 

CuLCrV accumulation in *B. tabaci* MED and *B. tabaci* MEAM1 and in their tissues differed significantly (*F*_5,54_ = 30.10; *p* < 0.001) (Figure 5B). More CuLCrV accumulated in *B tabaci* MEAM1 adults, MG, hemolymph, and PSG than in *B. tabaci* MED adults, MG, hemolymph and PSG. Similarly, SiGMV accumulation in *B. tabaci* MED and *B. tabaci* MEAM1 and their tissues differed significantly (*F*_5,54_ = 104.55; *p* < 0.001). More SiGMV accumulated in *B. tabaci* MEAM1 adults and tissues than in *B. tabaci* MED adults and tissues (Figure 5C).

### 3.5. Comparison of Capsid Protein Sequences of B. tabaci MED Transmissible and Non-Transmissible Begomoviruses

**Figure 6:**
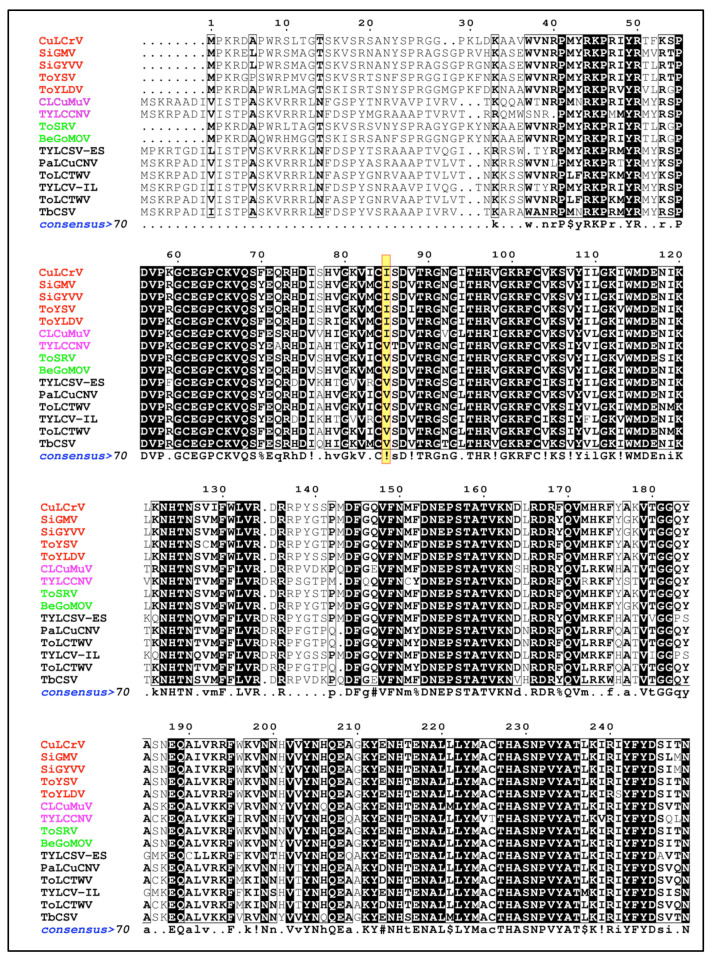
Comparison of capsid protein amino acid sequences of *B. tabaci* MED transmissible and non-transmissible begomoviruses. New-World non-transmissible (red); Old-World non-transmissible (magenta); New-World transmissible (green); and Old-World transmissible (black) begomoviruses. A consensus sequence was generated using MultAlin criteria: uppercase is identity, lowercase is consensus level > 0.5, ! is I or V, $ is L or M, % is F or Y, and # is N, D, Q, E, B or Z. At amino acid residue 85 (highlighted in yellow), all *B. tabaci* MED-non-transmissible viruses except for tomato yellow leaf curl China virus (TYLCCV) had valine, whereas *B. tabaci* MED-transmissible viruses had isoleucine.

An alignment of the capsid protein amino acid sequences of *B. tabaci* MED transmissible and non-transmissible begomoviruses did not reveal consistent mutations of amino acids at any location (Figure 6). At amino acid residue 85 located between two conserved regions in the capsid protein, GCEGPCKVQS and LYMACTHASN, all *B. tabaci* MED non-transmissible viruses except for tomato yellow leaf curl China virus (TYLCCV) had valine, whereas *B. tabaci* MED-transmissible viruses had isoleucine (Figure 6). 

## 4. Discussion

*Bemisia tabaci* MED has been in the United States since 2004, but it was restricted primarily to greenhouse-grown ornamentals [11]. However, *B. tabaci* MED has been recently detected outside of greenhouses in Florida and Georgia [12,13]. Recent reports of *B. tabaci* MED outside of the greenhouse have raised serious concerns on the implications for begomovirus epidemics in important vegetable crops in the southeastern United States. The current study compared the transmission efficiency of three economically important begomoviruses prevalent in the southeastern United States, viz., tomato yellow leaf curl virus (TYLCV), cucurbit leaf crumple virus (CuLCrV), and sida golden mosaic virus (SiGMV) between *B. tabaci* MEAM1 and *B. tabaci* MED. Both *B. tabaci* MED and *B. tabaci* MEAM1 were efficient vectors of the OW begomovirus, TYLCV. That was not the case for NW bipartite begomoviruses, CuLCrV and SiGMV. *Bemisia tabaci* MED was able to acquire and retain TYLCV, CuLCrV, or SiGMV after a 72 h AAP on TYLCV-infected tomato, CuLCrV-infected squash, or SiGMV-infected prickly sida plants, respectively. However, it was only able to inoculate TYLCV. In contrast, *B. tabaci* MEAM1 was able to acquire, retain, and inoculate non-infected tomato, squash, or prickly sida plants with TYLCV, CuLCrV, or SiGMV, respectively, after a 72 h AAP on the homologous infected hosts. Results from qPCR suggest that CuLCrV or SiGMV accumulated in the midgut of *B. tabaci* MED, but due to inefficient translocation from MG into hemolymph, CuLCrV or SiGMV failed to accumulate in the PSG, resulting in the inability to transmit CuLCrV or SiGMV. Taken together, results confirm that *B. tabaci* MED and *B. tabaci* MEAM1 differ in their ability to transmit NW bipartite begomoviruses, viz., CuLCrV and SiGMV.

A few other studies also have documented the differential transmission of begomoviruses by *B. tabaci* cryptic species. For instance, *B. tabaci* MEAM1 and *B. tabaci* MED differed in their ability to acquire and inoculate TYLCV isolate Sh2 reported from China [45]. Biotype A (New World 1) of *B. tabaci* has been reported as a better vector of chino del tomate virus than *B. tabaci* MEAM1 [87]. In the current study, *B. tabaci* MED accumulated and retained a higher amount of TYLCV than *B. tabaci* MEAM1, although this did not translate into differences in transmission. Similar transmission efficiency between the two cryptic species (MEAM1 and MED) appear to be associated with the ability of whiteflies to acquire the virus particles well above the threshold that would otherwise limit virus inoculation. Upon transfer to virus non-host cotton, TYLCV accumulation declined gradually in *B. tabaci* MED (5–9%/day) and *B. tabaci* MEAM1 (8–14%/day). The results of this study are consistent with previous findings on the gradual decline of TYLCV in viruliferous whiteflies once transferred to a virus non-host plant [45]. However, the TYLCV decline observed in the current study is greater than the 1–2% per day as reported earlier [88,89]. Su et al. (2013) reported that the rate of TYLCV decline in viruliferous *B. tabaci* MED on virus non-host cotton was associated with the presence or absence of an endosymbiont, *Hamiltonella* [89]. Yet, in the current study, the symbionts’ genera reported to affect transmission of begomoviruses, viz., *Hamiltonella*, *Arsenophonus,* and *Rickettsia* were present in both *B. tabaci* MEAM1 and *B. tabaci* MED populations (Supplementary Material, Table S3). Therefore, higher decline rates of TYLCV in *B. tabaci* MEAM1 and *B. tabaci* MED observed in the current study might be the result of differences in other factors such as experimental conditions, virus isolates, and/or whitefly populations.

CuLCrV or SiGMV accumulation in *B. tabaci* MEAM1 and *B. tabaci* MED was lower than TYLCV. This might be because of differences in virus accumulation in the host plants. TYLCV accumulation in tomato plants, estimated as copies per ng DNA, was higher than CuLCrV or SiGMV accumulation in squash and prickly sida plants, respectively (Supplementary Material, Figure S1). This correlation between virus accumulation in the host and in the vector has also been reported in multiple whitefly-begomovirus pathosystems [62,63,90,91,92]. After transfer to a virus non-host cotton, within a week, a sharp decline (90%) in CuLCrV or SiGMV accumulation was observed for *B. tabaci* MED, whereas in *B. tabaci* MEAM1, CuLCrV or SiGMV declined gradually (6–10%/day). Czosnek et al. (2002) compared the retention of TYLCV between vector (*B. tabaci*) and non-vector (*Trialeurodes vaporariorum*) whiteflies. They reported that *B. tabaci* was able to retain TYLCV for 15 days after a 4 d AAP on TYLCV-infected tomato, whereas TYLCV was undetectable in *T. vaporariorum* within a few hours post transfer to a virus non-host [93]. In the current study, *B. tabaci* MED acquired CuLCrV or SiGMV from infected plants after an AAP of 72 h and retained CuLCrV or SiGMV for much longer, albeit in lower amounts (>10-fold lower) than *B. tabaci* MEAM1. 

Some of the transmission differences between *B. tabaci* cryptic species have been associated with differences in their feeding habits. For example, *B. tabaci* MED has been documented to engage in prolonged and continuous feeding on TYLCV-infected plants than *B. tabaci* MEAM1, translating into increased acquisition and subsequent inoculation of TYLCV by *B. tabaci* MED [45]. In the current study, tomato, squash, and prickly sida plants were found to be equally susceptible to both *B. tabaci* MED and *B. tabaci* MEAM1. Therefore, it is possible that the differential transmission observed in this study might be the result of incompatible interactions between NW begomoviruses and *B. tabaci* MED. To further examine the differential transmission of CuLCrV or SiGMV by *B. tabaci* MEAM1 and *B. tabaci* MED, the current study, using qualitative (FISH) and quantitative (qPCR) techniques, traced the tropism of CuLCrV or SiGMV in *B. tabaci* MEAM1 and *B. tabaci* MED adults. Both viruses were able to traverse the midgut barrier of *B. tabaci* MEAM1 and *B. tabaci* MED adults. However, CuLCrV or SiGMV movement across the midgut of *B. tabaci* MED was less efficient in comparison with *B. tabaci* MEAM1. Lower-efficiency translocation across the *B. tabaci* MED midgut resulted in reduced virus accumulation in the hemolymph and consequently in the PSG, leading to an inability to transmit CuLCrV or SiGMV. Similar results have been reported for cotton leaf curl Multan virus (CLCuMuV), where inefficient movement of CLCuMuV across the MG of *B. tabaci* MEAM1 and *B. tabaci* MED adults in comparison with *B. tabaci* Asia II 1 resulted in reduced transmission of CLCuMuV in cotton [52]. None of the cotton plants inoculated by viruliferous *B. tabaci* MEAM1 and *B. tabaci* MED were infected with CLCuMuV, whereas 52% of cotton plants inoculated by viruliferous *B. tabaci* Asia II 1 were infected with the CLCuMuV [52]. The reduced translocation of CuLCrV or SiGMV across the midgut and accumulation in salivary glands was substantiated via FISH and qPCR. In the current study, qPCR analysis revealed that CuLCrV or SiGMV accumulation levels in the PSG of *B. tabaci* MEAM1 was ~ 100-fold more than in the PSG of *B. tabaci* MED. Additionally, FISH assay did not result in the localization of CuLCrV or SiGMV in the PSG of *B. tabaci* MED. The non-detection of FISH signals corresponding to CuLCrV or SiGMV in the PSG of *B. tabaci* MED might have been the consequence of reduced accumulation of CuLCrV or SiGMV in the PSG of *B. tabaci* MED than *B. tabaci* MEAM1. A previous study on TYLCV localization in *B. tabaci* MEAM1 using qPCR and FISH also demonstrated that qPCR allowed more sensitive and specific detection of TYLCV in whitefly tissues than FISH [94].

Capsid protein is the only known begomovirus-encoded protein that affects transmission by whiteflies [50]. Capsid protein amino acids between the two conserved regions CEGPCKVQS and LYMACTHASN have been reported to play a crucial role in determining begomovirus transmissibility by whiteflies [51,52,53]. Single or a combination of changes in the amino acid residues between these conserved regions has been shown to have a profound impact on begomovirus transmissibility by a whitefly cryptic species [55,95,96]. For instance, a double mutation from glutamine to proline and glutamine to histidine at amino acid resides 129 and 134, respectively, in the capsid protein of tomato leaf curl Sardinia virus (TYLCV-Sar) resulted in the loss of *B. tabaci* transmissibility [97]. Similarly, non-transmissible abutilon mosaic virus (AbMV) isolate was readily transmitted by the M biotype of *B. tabaci* after substitution of glutamine with lysine, histidine with glutamine, and leucine with methionine at amino acid residues at 124, 149 and 174, respectively [96]. Further, Pan et al., while examining the key amino acid residues responsible for differential transmission of squash leaf curl China virus (SLCCNV), found that a threonine to serine substitution at residue 147 altered the SLCCNV transmission by *B. tabaci* Asia 1 and *B. tabaci* Asia II [95]. An alignment of the amino acid sequences of the capsid protein of *B. tabaci* MED transmissible and non-transmissible begomoviruses did not reveal any consistent patterns in this study. However, at amino acid residue location 85, all *B. tabaci* MED non-transmissible viruses except for tomato yellow leaf curl China virus (TYLCCV) had valine, whereas *B. tabaci* MED-transmissible viruses had isoleucine (Figure 6). Whether this change is just a random occurrence or if it plays any role in determining the interactions between begomoviruses and *B. tabaci* MED is not clear and warrants further investigation. These results suggest that the inability of *B. tabaci* MED to transmit the two NW bipartite viruses could possibly be due to random mutations within the capsid protein or other vector determinants.

## 5. Conclusions

The current study showed that the circulative movement of OW (TYLCV) and NW (CuLCrV and SiGMV) begomoviruses within *B. tabaci* MED differed in their ability to cross the midgut barrier. During circulative movement within *B. tabaci*, begomoviruses interact with many vector receptors, immune proteins, and/or symbionts’ proteins located at MG, hemolymph, and PSG [48,98,99,100]. For instance, two *B. tabaci* MEAM1 midgut proteins, viz., BtCUBN and amnionless (BtAMN), form a BtCubam receptor complex that binds to the capsid protein of TYLCV, resulting in clathrin-mediated endocytosis of TYLCV into whitefly midgut cells [101]. In the present study, both *B. tabaci* MED and *B. tabaci* MEAM1 efficiently transmitted TYLCV, but only *B. tabaci* MEAM1 was able to transmit CuLCrV or SiGMV. Therefore, it is very likely that factors required for efficient circulation of TYLCV within whiteflies are present in both *B. tabaci* MED and *B. tabaci* MEAM1. However, factors required for efficient circulation of CuLCrV or SiGMV were present only in *B. tabaci* MEAM1. Thus, it is possible that different begomoviruses interact with different whitefly transmission determinants and these factors could differ between cryptic species. Currently, these whitefly factors remain incompletely understood and warrant further investigation. Moreover, at this time it is not clear whether the longer association between NW begomoviruses with *B. tabaci* MEAM1 (30–40 years) in comparison with *B.tabaci* MED (~15 years) provided an opportunity for the NW begomoviruses and *B. tabaci* MEAM1 to coevolve favoring transmission, or whether these differential interactions are a result of innate transmission determinants. From a limited number of studies conducted, it seems that *B. tabaci* MED readily transmits OW begomoviruses, whereas *B. tabaci* MEAM1 seems to be an effective vector of both OW and NW begomoviruses. The establishment of *B. tabaci* MED in the United States can have severe impacts on OW begomovirus epidemics, such as TYLCV in tomato production. However, similar trends may not be observed for NW begomoviruses, such as CuLCrV and SiGMV, with the caveat being that this study was conducted only with these two NW begomoviruses and under controlled conditions.

## Figures and Tables

**Table 1:** Primers used for detection and quantitation of DNA-A of TYLCV, CuLCrV, and SiGMV.

Virus	GenBank Accession Numbers	Name	Sequence (5′–3′)	Product Size (bp)
TYLCV	KY971372	TYLC-C2-For	GCAGTGATGAGTTCCCCTGT	102
		TYLC-C2-Rev	CCAATAAGGCGTAAGCGTGT	
		C2-1159	CTAGATCCACTGCTCTGATTACA	694
		C2-1853	TCATTGATGACCTAGCAAAG	
CuLCrV	AF224760	CuLCrV-QF	CCTCAAAGGTTTCCCGCTCT	110
		CuLCrV-QR	CCGATAGATCCTGGGCTTCC	
		3FAC3	TTTATATCATGATTTTCGAGTACA	525
		5RAC1	AAAATGAAAGCCTAAGAGAGTGGA	
SiGMV	MK387701	SiGMV-QF	CTCAAAGGTTAGCCGCAACG	114
		SiGMV-QR	CGGTAGATCCTGGGCTTCCT	
		SiGMVF	TTCTCCTCGTGCAGGTAGTG	574
		SiGMVR	ACTTGCCAGCCTCTTGATGA	

**Table 2:** *Bemisia tabaci* MED transmissible and non-transmissible begomoviruses.

Sr. No.	Virus	Transmission Efficiency (No. of Viruliferous Whitefly Used Plant)	Accession Number	Type ^a^	Reference
1	Cucurbit leaf crumple virus (CuLCrV)	0% (100)	AF256200	NW (bipartite)	Current study
2	Sida golden mosaic virus (SiGMV)	0% (100)	MK387701	NW (bipartite)	Current study
3	Sida golden yellow vein virus (SiGYVV)	0% (50)	HQ822123	NW (bipartite)	[71]
4	Bean golden mosaic virus (BGMV)	100% (10)	NC_004042	NW (bipartite)	[72]
5	Tomato severe rugose virus (ToSRV)	10% (15)	MG837738	NW (bipartite)	[73]
6	Tomato yellow spot virus (ToYSV)	0% (15)	DQ336350	NW (bipartite)	[73]
7	Tomato leaf deformation virus (ToYLDV)	0% (50)	NC_014510	NW (monopartite)	[71]
8	Tomato yellow leaf curl China virus (TYLCCNV)	0% (10)	AJ319675	OW (monopartite)	[53]
9	Tobacco curly shoot virus (TbCSV)	75% (5)	AJ420318	OW (monopartite)	[74]
10	Tomato yellow leaf curl Sardinia virus (TYLCSV-ES)	40% (2)	Z25751	OW (monopartite)	[75]
11	Tomato yellow leaf curl virus Israel strain (TYLCV)	50% (2)	KY965923	OW (monopartite)	[45]
12	Cotton leaf curl Multan virus (CLCuMuV)	0% (10)	KP762786	OW (monopartite)	[76]
13	Papaya leaf curl China virus (PaLCuCNV)	~60% (10)	AM691554	OW (monopartite)	[77]
14	Tomato leaf curl Taiwan virus (ToLCTWV)	45% (5)	DQ866122	OW (monopartite)	[78]
17	Tomato leaf curl New Delhi virus (ToLCNDV)-ES	100% (25–30)	KF749224	OW (bipartite)	[79]
18	Sri Lankan cassava mosaic virus (SLCMV)	63.3% (10)	MH891840	OW (bipartite)	[80]
19	Tomato yellow leaf curl Thailand virus (TYLCTHV)	50% (5)	GU723742	OW (bipartite)	[78]
20	Ramie mosaic virus (RaMoV)	~100% (2)	KU522485	OW (bipartite)	[81]

^a^ NW = New World; OW = Old World.

**Table 3:** *Bemisia tabaci* MEAM1 and *B. tabaci* MED life history parameters on tomato, squash, or prickly sida plants.

Plant Type	Whitefly Type	N ^a^	Percent Egg Survival		Egg-Adult ^b^		N ^c^	Fecundity	
Tomato	MEAM1	45	80 ± 3.80 73 ± 10.20	*χ*^2^_1,88_ = 2.26; *p* = 0.21	21 (18–29)	*χ*^2^_1,58_ = 4.35; *p* = 0.23	30	72 ± 7.20	*F*_1,58_ = 3.59 *p* = 0.16
	MED	45			21 (17–30)		30	82 ± 5.30	
Squash	MEAM1	45	78 ± 8.00	*χ*^2^_1,88_ = 3.31; *p* = 0.32	21 (19–30)	*χ*^2^_1,59_ = 3.41; *p* = 0.52	30	68 ± 13.50	*F*_1,58_ = 1.09 *p* = 0.41
	MED	45	69 ± 9.70		22 (18–28)		30	53 ± 5.30	
Prickly sida	MEAM1	45	56 ± 4.40	*χ*^2^_1,88_ = 7.26; *p* = 0.08	23 (19–32)	*χ*^2^_1,42_ = 6.23; *p* = 0.32	30	34 ± 9.20	*F*_1,58_ = 4.76 *p* = 0.09
	MED	45	53 ± 10.20		24 (19–33)		30	36 ± 2.90	

^a^ Number of eggs monitored to adulthood. ^b^ Median developmental time (days) from egg to adult with range in parentheses. ^c^ Number of *B. tabaci* pairs used for fecundity studies.

## Data Availability

Not applicable.

## Supplementary Materials

The following supporting information can be downloaded at: https://www.mdpi.com/article/10.3390/v14051104/s1. Table S1: Validation of insects and plant samples surface sterilization; Table S2: Infection or non-infection of cotton with TYLCV, CuLCrV, or SiGMV; Table S3: Primary and secondary endosymbionts within *B. tabaci* MEAM1 and *B. tabaci* MED populations; Figure S1: TYLCV, CuLCrV, or SiGMV accumulation in tomato, squash, or prickly sida.
