# Supplementary material for: Differential Transmission of Old and New World Begomoviruses by Middle East-Asia Minor 1 (MEAM1) and Mediterranean (MED) Cryptic Species of Bemisia tabaci
Source: Viruses. 2022 May 20;14(5):1104. doi: 10.3390/v14051104 (PMC9146840; doi:10.3390/v14051104)
Supplement: Supplementary file 1 [file viruses-14-01104-s001.zip › viruses-1718261-supplementary.pdf]

viruses

Article

Differential Transmission of Old and New World Begomoviruses by Middle East-Asia Minor 1 (MEAM1) and Mediterranean (MED) Cryptic Species of *Bemisia tabaci*

Saurabh Gautam [1], Habibu Mugerwa [1], James W. Buck [2], Bhabesh Dutta [3], Tim Coolong [4], Scott Adkins [5] and Rajagopalbabu Srinivasan [1,*]

[1] Department of Entomology, University of Georgia, 1109 Experiment Street, Griffin, GA 30223, USA; sg37721@uga.edu (S.G.); habibu.mugerwa@uga.edu (H.M.)
[2] Department of Plant Pathology, University of Georgia, 1109 Experiment Street, Griffin, GA 30223, USA; jwbuck@uga.edu
[3] Department of Plant Pathology, University of Georgia, 3250 Rainwater Road, Tifton, GA 31793, USA; bhabesh@uga.edu
[4] Department of Horticulture, University of Georgia, 3250 Rainwater Road, Tifton, GA 31793, USA; tcoolong@uga.edu
[5] United States Department of Agriculture-Agricultural Research Service, U.S. Horticultural Research Laboratory, Fort Pierce, FL 34945, USA; scott.adkins@usda.gov
* Correspondence: babusri@uga.edu

Methods

S.1 Validation of surface sterilization

Viruliferous *Bemisia tabaci* MEAM1 (5-10 days old) were obtained by providing an AAP of 72h on TYLCV-infected tomato plants. Following the AAP, 10 whiteflies were randomly selected and tested for the presence of virus using endpoint PCR. Viruliferous whiteflies were released on two-true leaf stage cotton plants and were allowed to feed and secrete honeydew for next five days (inoculation access period, IAP). Following IAP, whiteflies were aspirated and plants were transferred to new insect free cage. One hundred newly emerged (upto 48h old) non-viruliferous whiteflies were released on the cotton plants contaminated with honeydew secreted by viruliferous *B. tabaci* MEAM1. After 72h, whiteflies were aspirated from plants, and all leaves were excised. Collected whiteflies were stored at -20°C. After 24h, whiteflies were removed from the freezer and divided into six groups with 10 whiteflies each. Out of the six groups, three were surface sterilized. Total DNA from individual surface-sterilized or non-surface sterilized whiteflies was extracted using InstaGene matrix and subject to endpoint PCR using the conditions described in the manuscript. Excised cotton leaves were chopped into 2x2 cm^2. Total DNA from ten surface-sterilized or non-surface sterilized chopped leaf samples was extracted using GeneJET Plant Genomic Purification Kit (ThermoFisher Scientific, MA) and subject to endpoint PCR using conditions described in the manuscript.

S. 2 Infection or non-infection of cotton with TYLCV, CuLCrV, or SiGMV

Viruliferous *B. tabaci* MEAM1 were obtained by providing whiteflies a 72h AAP on respective infected plants (TYLCV-infected tomato, CuLCrV-infected squash, or SiGMV-infected prickly sida, and). Viruliferous *B. tabaci* MEAM1 (~100/plant) were released on two-true-leaf stage cotton plants. As a positive control, 100 respective viruliferous whiteflies/plant were also released on tomato, squash or prickly sida. Plants were then held in the greenhouse (25–30°C, 14h L:10h D) in insect-proof cages. Four weeks later, the topmost leaf sample (100 mg) was excised and surface-sterilized using a six-step surface sterilization protocol (in manuscript). Surface-sterilized leaf tissues were used for DNA extraction. Total genomic DNA was extracted with the GeneJET Plant Genomic Purification Kit (ThermoFisher Scientific, MA, USA). Virus infection status in plants was determined

Citation: Gautam, S.; Mugerwa, H.; Buck, J.W.; Dutta, B.; Coolong, T.; Adkins, S.; Srinivasan, R. Differential Transmission of Old- and New-World Begomoviruses by Middle East-Asia Minor 1 (MEAM1) and Mediterranean (MED) Cryptic Species of *Bemisia tabaci*. *Viruses* **2022**, *14*, 1104. http://doi.org/10.3390/v14051104

Academic Editor: Patrick Bron

Received: 24 April 2022
Accepted: 18 May 2022
Published: 20 May 2022

Publisher's Note: MDPI stays neutral with regard to jurisdictional claims in published maps and institutional affiliations.

Copyright: © 2022 by the authors. Licensee MDPI, Basel, Switzerland. This article is an open access article distributed under the terms and conditions of the Creative Commons Attribution (CC BY) license (https://creativecommons.org/licenses/by/4.0/).

through end-point PCR using conditions described in the manuscript. Each treatment had 10 replications.

S.3 Primary and secondary endosymbionts within B. tabaci MEAM1 and B. tabaci MED populations

The molecular detection and identification of primary and secondary endosymbionts within *B. tabaci* MEAM1 and B. *tabaci* MED populations followed the protocols described earlier by Ghosh et al. 2015 [101]. Briefly, 15 whiteflies were randomly collected from *B. tabaci* MEAM1 or *B. tabaci* MED colonies and divided into three groups with 5 whiteflies in each. The total nucleic acid was extracted from each group in 500 µl InstaGene Matrix containing six percent Chelex resin (Bio-Rad, Hercules, CA) following the manufacturer's protocol. For endpoint PCR, extracted total genomic DNA (100 ng), 25 µL of GoTaq Green Mastermix buffer (2X) (Promega, Madison, WI)**,** 0.5µM of forward and reverse primers and nuclease-free water were combined for a final volume of 50 µl. The PCR reactions were conducted using a thermocycling protocol of 94 °C for 3 min followed by 40 cycles of 30 sec at 94 °C, 30 sec at 56-60 °C, and 90 sec at 72 °C, with a final extension of 5 min at 72 °C (Table S1). The PCR amplicons were sequenced in both directions (Eurofin genomics,Louisville, KY). The obtained sequences were subjected to a BLASTn search in NCBI to confirm their identity.

Table S1. Endosymbioant screening in *B. tabaci* MEAM1 and *B. tabaci* MED.

Symbiont genera	Target gene	Primer Name	Sequence (5′→3′)	Annealing temperature	Amplicon length
Portiera (P)	*Portiera* 16S rDNA	28F<br>1098R	TGCAAGTCGAGCGGCATCAT<br>AAAGTTCCCGCCTTATGCGT	56°C	1050bp
Arsenophonus (S)	*Arsenophonus* 23S rDNA	Ars23S-1<br>Ars23S-2	CGTTTGATGAATTCATAGTCAAA<br>GGTCCTCCAGTTAGTGTTACCCAAC	58°C	750bp
Rickettsia (S)	Rickettsia 16S rDNA	Rb-F<br>Rb-R	GCTCAGAACGAACGCTATC<br>GAAGGAAAGCATCTCTGC	58°C	960bp
Wolbachia (S)	*Wolbachia* 16S rDNA	Wol16S- F<br>Wol16S- R	CGGGGGAAAAATTTATTGCT<br>CCCCATCCCTTCGAATAGGTAT	58°C	730bp
Cardinium (S)	*Cardinium* 16S rDNA	Card-F<br>Card-R	TAGACACACACGAAAGTTCATGT<br>TAGACACACACGAAAGTTCATGT	58°C	650bp
Hamiltonella (S)	*Hamiltonella* 16S rDNA	Hb-F<br>Hb-R	TGAGTAAAGTCTGGGAATCTGG<br>AGTTCAAGACCGCAACCTC	56°C	730bp
Fritschea (S)	*Fritschea* 23S rDNA	Frit-F<br>Frit-R	GAGTTTGATCATGGCTCAGATTG<br>GCTCGCGTACCACTTTAAATGGCG	60°C	630 bp
Hemipteriphilus (S)	*Hemipteriphilus* 16S rRNA	Hem-F:<br>Hem-R:	GCTCAGAACGAACGCTRKC<br>TTCGCCACTGGTGTTCCTC	58°C	670 bp

Results

S.1 Validation of surface sterilization

About 3.33% of initially non-viruliferous *B. tabaci* MEAM1 tested positive for TYLCV in non-surface sterilized samples. However, none of the surface sterilized non-viruliferous whiteflies tested positive for the virus (Table S2). Thirty percent of the cotton leaf samples tested positive for TYLCV in non-surface sterilized samples. However, none of the surface sterilized leaf samples tested positive for the TYLCV (Table S2).

Table S2. Validation of insects and plant samples surface sterilization.

Sr. No.		Non-surface sterilized			Surface sterilized		
		**Rep1**	**Rep2**	**Rep3**	**Rep1**	**Rep2**	**Rep3**
1	Whiteflies	0/10	1/10	0/10	0/10	0/10	0/10
		**Rep1**			**Rep1**		
2	Cotton	3/10			0/10		

S.2 Infection or non-infection status of cotton with TYLCV, CuLCrV, or SiGMV

None of the cotton plants subjected to whiteflies that had acquired TYLCV, CuLCrV, or SiGMV tested positive for the virus. However, 100% of tomato were positive for TYLCV, 70% of squash plants were tested positive for CuLCrV, and 90% of prickly sida plants were tested positive for SiGMV.

S.3 Primary and secondary endosymbionts within B. tabaci MEAM1 and B. tabaci MED populations

Both *B. tabaci* cryptic species (MEAM1 and MED) harbored the primary obligatory symbiont *Portiera.* Facultative secondary symbionts such as *Arsenophonus, Rickettsia,* and *Hamiltonella* were present in both *B. tabaci* MEAM1 and *B. tabaci* MED populations (Table S3). However, secondary symbionts such as *Wolbachia, Cardinium, Fritschea,* and *Hemipteriphilus* could not be detected in the tested individuals of both *B. tabaci* MEAM1 and *B. tabaci* MED.

Table S3. Prevalence of endosymbionts in *B. tabaci* MEAM1 and *B. tabaci* MED populations.

Symbiont genera	Whitefly type	Presence/absence	Accession No.
Portiera (P)	MEAM1	+	OK576636
	MED	+	OK665696
Arsenophonus (S)	MEAM1	+	OK598060
	MED	+	OK665698
Rickettsia (S)	MEAM1	+	OK576635
	MED	+	OK665695
Wolbachia (S)	MEAM1	-	
	MED	-	
Cardinium (S)	MEAM1	-	
	MED	-	
Hamiltonella (S)	MEAM1	+	OK576637
	MED	+	OK665697
Fritschea (S)	MEAM1	-	
	MED	-	
Hemipteriphilus (S)	MEAM1	-	
	MED	-	

+ presence confirmed via PCR followed by sequencing; - not detectable in PCR.

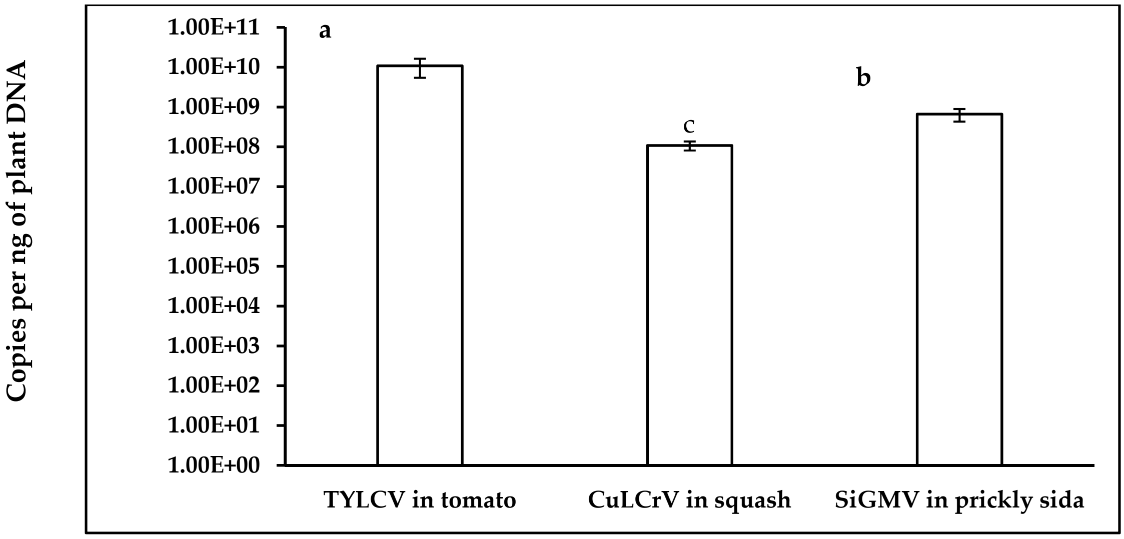

Figure S1. TYLCV, CuLCrV, or SiGMV accumulation in tomato, squash, or prickly sida.

Bars with standard errors represent average number of TYLCV, CuLCrV, or SiGMV copies per ng DNA in tomato, squash, or prickly sida plants, respectively. Y-axis on panel is represented in logarithmic scale. Significant differences between means were separated with Tukey's HSD posthoc test at $\alpha = 0.05$
